# Cross-phenotype association tests uncover genes mediating nutrient response in *Drosophila*

**DOI:** 10.1186/s12864-016-3137-9

**Published:** 2016-11-04

**Authors:** Christopher S. Nelson, Jennifer N. Beck, Kenneth A. Wilson, Elijah R. Pilcher, Pankaj Kapahi, Rachel B. Brem

**Affiliations:** 1 Buck Institute for Research on Aging, 8001 Redwood Blvd., Novato, CA 94947 USA; 2 Davis School of Gerontology, University of Southern California, Los Angeles, CA USA; 3 Department of Urology, University of California, San Francisco, CA USA; 4 Department of Plant and Microbial Biology, University of California, Berkeley, Berkeley, CA USA

**Keywords:** GWAS, *Drosophila*, Nutrition, Obesity, Dietary restriction

## Abstract

**Background:**

Obesity-related diseases are major contributors to morbidity and mortality in the developed world. Molecular diagnostics and targets of therapies to combat nutritional imbalance are urgently needed in the clinic. Invertebrate animals have been a cornerstone of basic research efforts to dissect the genetics of metabolism and nutrient response. We set out to use fruit flies reared on restricted and nutrient-rich diets to identify genes associated with starvation resistance, body mass and composition, in a survey of genetic variation across the *Drosophila* Genetic Reference Panel (DGRP).

**Results:**

We measured starvation resistance, body weight and composition in DGRP lines on each of two diets and used several association mapping strategies to harness this panel of phenotypes for molecular insights. We tested DNA sequence variants for a relationship with single metabolic traits and with multiple traits at once, using a scheme for cross-phenotype association mapping; we focused our association tests on homologs of human disease genes and common polymorphisms; and we tested for gene-by-diet interactions. The results revealed gene and gene-by-diet associations between 17 variants and body mass, whole-body triglyceride and glucose content, or starvation resistance. Focused molecular experiments validated the role in body mass of an uncharacterized gene, *CG43921* (which we rename *heavyweight*), and previously unknown functions for the diacylglycerol kinase *rdgA*, the huntingtin homolog *htt*, and the ceramide synthase *schlank* in nutrient-dependent body mass, starvation resistance, and lifespan.

**Conclusions:**

Our findings implicate a wealth of gene candidates in fly metabolism and nutrient response, and ascribe novel functions to *htt*, *rdgA*, *hwt* and *schlank*.

**Electronic supplementary material:**

The online version of this article (doi:10.1186/s12864-016-3137-9) contains supplementary material, which is available to authorized users.

## Background

Obesity is a worldwide epidemic and among the leading causes of morbidity and mortality in the US, increasing susceptibility to chronic pathologies that impact many body systems [1]. Against a backdrop of landmark advances in dissections of the basic biology of metabolism [2], geneticists continue to search for novel genes that modulate body composition and diet responses, as potential targets for drug development. Such factors have emerged from mapping studies in human populations [3] and in mice reared on a high-fat diet [4–6], though only a handful have been validated at the single-gene level [3]. As a complement to mammals, invertebrate model organisms offer a far quicker route to the discovery and validation of nutrient response genes. In the fruit fly, classic mutagenesis screens have enabled landmark discoveries of obesity and body composition genes conserved in vertebrates [7–12]. These screens have been applied to one qualitative, easily-scored trait at a time. Consequently, geneticists have had an incomplete understanding of how multiple aspects of metabolism are coordinated in response to the organism’s changing needs, even for the well-studied insulin [13, 14] and target of rapamycin (TOR) [15] pathways in the fly and other model organisms.

The advent of the *Drosophila* Genetic Reference Panel (DGRP) enables the mapping of natural phenotypic variation to DNA sequence in flies with an association paradigm [16, 17]. Genome-wide association studies (GWAS), by using populations in which many natural genetic variants are segregating at once, can require orders of magnitude less phenotyping effort than systematic screens perturbing each gene in turn. As such, GWAS makes possible the genetic dissection of traits that require assays too nuanced or time-consuming for mutagenesis studies, including many aspects of metabolism and dietary response. And alongside its use in gene discovery, GWAS also reveals the genetic architecture of natural variation in a trait of interest. These strengths have motivated previous studies focused on metabolic rate and body size and composition in the fly, yielding a number of intriguing candidate genes and polymorphisms [16, 18–22].

A potential strategy to boost power in GWAS is the use of cross-phenotype association tests, to search for cases in which a genetic variant associates with multiple traits at once [23]. The recent success of principal component-based methods for this purpose in the fly [24] has led to a first set of candidate cases in which master regulatory loci associate jointly with multiple facets of metabolism. We reasoned that any such candidate would serve as a high-value candidate for experimental validation. We also expected that association tests for gene-by-diet interactions, which have not as yet been implemented in the fly [19, 21, 25, 26], could be harnessed to reveal novel genetic determinants of metabolism. We thus set out to use cross-phenotype association tests in the DGRP, along with single-phenotype GWAS approaches, to generate testable hypotheses about genes underlying metabolic traits and the response to changing diet.

## Results

We surveyed natural genetic variation across genetically distinct fly lines in metabolic behaviors and their dependence on dietary yeast restriction. Dietary yeast levels are known to modulate fly body composition, stress resistance, and lifespan in laboratory strains [27–30]. We reared 171–181 DGRP lines on two diets (*ad libitum*, AL, 5 % yeast; dietary restriction, DR, 0.5 % yeast), using non-virgin females to avoid effects of mating status on diet–responsive phenotypes [31]. In each, we measured wet body mass, resistance to acute starvation, and whole-body triglyceride and glucose levels (Additional file 1: Figure S1). As expected [32–36], on average DR treatment was associated with an increase in all traits except wet body mass, which decreased in DR flies relative to AL flies (Additional file 1: Figure S1). Phenotype values of a given strain reared on the two diets were correlated (*R* ^2^ = 0.53-0.69; Additional file 1: Table S1), indicating a sizeable genetic contribution to phenotype, independent of diet. Indeed, heritabilities ranged from modest to high for the GWAS phenotypes (47–93 %; Additional file 1: Table S2). For most traits, the ratio of the respective values measured in flies reared on the AL and DR diets was also highly heritable (Additional file 1: Table S2).

### GWAS of metabolic traits

To begin to identify the genetic basis of variation across DGRP lines in metabolic behaviors, we tested each trait in turn for association to each of the 1.9 million variants segregating in the population with minor allele frequency ≥5 %. Our association paradigm used a linear regression method that included terms in the model for the effect of genotype at a given locus, its interaction with diet, and diet itself, and we corrected for multiple testing via permutation. At an experiment-wise threshold for significance corresponding to false discovery rate ≤ 10 %, we mapped diet-independent effects of one polymorphic locus, *nimb3*, to measurements of resistance of acute starvation, and another locus, *14*-*3*-*3 epsilon*, to whole-body triglyceride content (Table 1). The top-scoring polymorphisms in these genes explained 18–20 % of the genotypic variance in their respective phenotypes (Table 1). Together, these association results provided a first line of evidence that our GWAS could identify genes that govern variation in fly metabolism.

**Table 1:** Variants associated with metabolic traits

Screen^a^	Phenotype(s)^b^	Test^b^	Chr^c^	Position^c^	Gene^c^	% Var^d^	*p* ^e^	FDR^f^
MAF ≥ 5 %	starvation resistance	genotype	2L	13967604	*nimB3*	18 %	3.70E-13	0 %
MAF ≥ 5 %	triglycerides	genotype	3R	14072066	*14*-*3*-*3 ε*	20 %	1.29E-14	10 %
MAF ≥ 5 %	starvation resistance and triglycerides on AL	SMAT	3L	11418293	*CG7560*	8 %	8.88E-16	0 %
MAF ≥ 5 %	starvation resistance and triglycerides on AL	SMAT	2R	16952034	*Cht12*	6 %	6.18E-11	10 %
MAF ≥ 5 %	starvation resistance and mass on AL	SMAT	3R	19635620	*EIF4G2*	8 %	1.71E-13	10 %
MAF ≥ 5 %	starvation resistance and mass on AL	SMAT	3R	24549344	*htt*	12 %	2.78E-12	10 %
MAF ≥ 5 %	starvation resistance and mass on DR	SMAT	X	6152605	*schlank*	3 %	4.44E-16	0 %
MAF ≥ 5 %	starvation resistance and mass on DR	SMAT	X	6152619	*schlank*	3 %	4.44E-16	0 %
MAF ≥ 5 %	starvation resistance and mass on DR	SMAT	X	6152558	*schlank*	2 %	6.66E-16	0 %
MAF ≥ 5 %	starvation resistance and mass on DR	SMAT	X	6152586	*schlank*	2 %	8.88E-16	0 %
MAF ≥ 25 %	body mass	genotype	X	12430723	*CG43921*	15 %	3.84E-13	0 %
MAF ≥ 25 %	glucose	interaction	3R	8877613	*pic*	4 %	5.94E-3	7 %
MAF ≥ 25 %	glucose	interaction	3R	8877592	*pic*	4 %	6.72E-3	7 %
MAF ≥ 25 %	glucose	interaction	3R	8877227	*CG7966*	5 %	6.75E-3	7 %
MAF ≥ 25 %	glucose	interaction	3R	8877378	*CG7966*	5 %	8.69E-3	8 %
MAF ≥ 25 %	starvation	genotype	2L	13967604	*nimB3*	18 %	3.70E-13	0 %
MAF ≥ 25 %	starvation resistance and mass on DR	SMAT	X	8903670	*rdgA*	15 %	1.27E-08	10 %
MAF ≥ 25 %	starvation resistance and triglycerides on DR	SMAT	3R	4279209	*CG43462*	8 %	1.29E-09	10 %

^a^Results from association scans across markers meeting the indicated criterion. For results from the scaled multiple-phenotype association test (SMAT) applied to variants at frequency of ≥5 %, only human disease orthologs were tested

^b^The single phenotype whose measurements in animals reared on both diets were tested in a linear model with terms for diet, genotype, and the interaction between the two; or the pair of phenotypes whose measurements in animals reared on the indicated diet were tested via SMAT. DR, dietary restriction; AL, *ad libitum*

^c^Chromosome segment, position (in *D. melanogaster* genome version R5 coordinates), and gene in which the associating marker lay

^d^Percent phenotypic variation explained by the indicated polymorphism. For pairs of phenotypes, the value reports results for the single phenotype with the highest % variance explained

^e^Nominal association *p*-value

^f^False discovery rate at the association *p*-value according to permutation analysis

We used the results from each genome-wide scan as input into tests of groups of genes of common function for enriched association signal, using GOglm [37]. Emerging from this analysis were eight GO terms whose genes tended to associate with different aspects of metabolism (Additional file 1: Table S3). Most notable was the enrichment of association signal, for body mass and glucose levels, in sensory perception and antennal morphogenesis genes, suggesting the possibility that some variants modulating these traits act by tuning animals’ perception of food [38–40].

### Cross-phenotype association mapping

We reasoned that a complementary strategy to mine our panel of metabolic trait measurements in DGRP lines would be to test a given variant for association with multiple traits at once. For this purpose, we used each pair of phenotypes in turn (from among our set of four phenotypes in animals reared on each diet; Additional file 1: Figure S1) as input into the multiple-phenotype association test (SMAT; [41]). Genome-wide scans with this paradigm were not sufficiently well-powered to map loci at reasonable false discovery rates after permutation-based multiple testing correction (see Methods and Additional file 1: Table S4). To improve power, and motivated by the clinical interest in human diseases where nutrition is a risk factor, we developed a test pipeline specialized to fly homologs of a broad range of human disease genes (as curated by Online Mendelian Inheritance in Man [42]; see Methods). Our rationale was that any genes of this set, if mapped in our association calculations, would likely be only a subset of the full complement of loci causal for the respective trait, but would be of particular interest as candidates for translational applications. Scans of human disease loci using SMAT attained power in a number of trait pairs, when considered independently (Additional file 1: Table S4) and when corrected for multiple testing across pairs (see Methods). Emerging from the results was strong signal for six polymorphisms jointly associating with starvation resistance and body mass, in DR flies and, separately, in flies reared on the AL diet (Table 1). These polymorphisms were in eIF4G2, a paralog of the nutrient-sensing factor eIF4G [43]; the ceramide synthase gene *schlank*; and the *Drosophila* homolog of huntingtin, the gene underlying human Huntington’s disease (Table 1). These associations explained 8–12 % of the genetic variance in starvation resistance or body mass (Table 1). Cross-phenotype mapping also found associations between starvation resistance and triglyceride levels and two loci (Table 1): *CG7560*, an FAD-linked oxidoreductase involved in methionine metabolism [44], and *Cht12*, a chitinase involved in cuticle formation [45]. Tests for gene sets with enriched association signal in cross-phenotype tests revealed association between starvation resistance and glucose content and genes annotated in ventral cord development (Additional file 1: Table S3), raising the possibility that these characters could be affected by variants that act in the central nervous system, plausibly via locomotor hyperactivity during starvation [38–40]. These data established our cross-phenotype association tests as a useful tool in the mapping of genotype to phenotype for metabolic traits.

### A new role for *schlank* in nutrient response and lifespan in adult flies

**Fig. 1:**
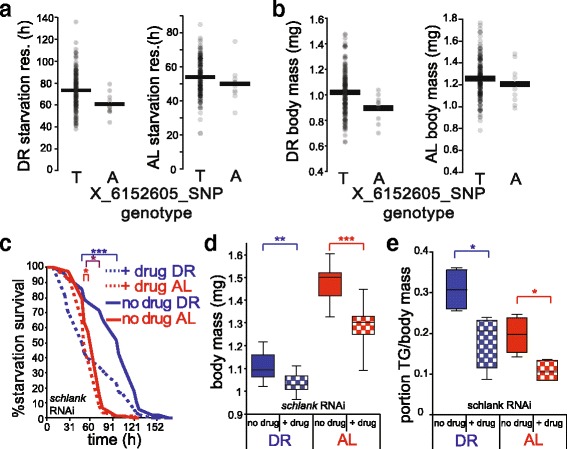
The ceramide synthase *schlank* governs body mass, starvation, and lifespan. **a** and **b** Trait association to *schlank* genotype in the DGRP. In a given panel, each point reports phenotype measurements (*y*-axis) from one DGRP strain homozygous for the *schlank* allele indicated on the *x*-axis. Each black horizontal bar reports the mean phenotype across all strains harboring the indicated allele. **c**-**e** Validation of *schlank* function. **c** Each trace reports survival of a strain harboring a *schlank* RNAi construct and a GAL4-GeneSwitch (GS) activator under the *daughterless* promoter, pre-treated with RU486 (+ drug, dotted lines) or vehicle (no drug, solid lines) for 10 days on the indicated diet and then switched to acute starvation media. **d** Each column reports body mass per fly of the indicated strain, with genotypes as in **c** (*n* = 2 populations of 15 flies per condition). **e** Each column reports whole body triglyceride levels per fly of the indicated strain, with genotypes as in **c** (*n* = 1 population of 15 flies per condition). For **d** and **e** top, middle, and bottom horizontal bars of a given vertical box denote the respective quartiles over batches and/or technical replicates, and the top and bottom short horizontal bars report minimum and maximum, respectively. AL, *ad libitum* diet; DR, dietary restriction. *, *p* < 0.05, **, *p* < 0.01, ***, *p* < 10^-3^, ****, *p* <10^-7^, *****, *p* < 10^-15^. Red asterisks denote significance of the effect of the genetic perturbation in animals on AL food, blue denotes significance in animals on DR food, and purple denotes significance of the interaction between diet and genetic perturbation. For strain details see Additional file 1: Table S6

Among our cross-phenotype association hits, as a first test of functional validation we focused on *schlank*, given its known role as a determinant of lipid accumulation in fly larvae [46] and, in yeast, as a determinant of lifespan [47]. The association between intronic variants in *schlank* and starvation resistance and body mass reached experiment-wide significance in DR flies (Table 1) and was also apparent in flies on the AL diet (Fig. 1a-b). Since loss of *schlank* function compromises viability beyond the larval stage [46], to investigate its role in metabolic traits during adulthood we crossed a stock ubiquitously expressing the GeneSwitch (GS) regulator, which is inducible upon treatment with RU486 [48], to a TRiP background stock [49] harboring a GAL4-controlled *schlank* RNAi transgene. In the progeny, upon induction we confirmed *schlank* knockdown by qRT-PCR (Additional file 1: Figure S2). We first focused on acute starvation resistance, which increases in wild-type flies reared under chronic nutrient deprivation in a manner dependent on changes in fat metabolism [36]. Strikingly, *schlank* knockdown during adulthood completely abrogated this advantage of the DR treatment: starvation resistance in knockdown flies reared on DR was 59 % of wild-type controls, on par with that of animals reared under AL (Fig. 1c). Further testing revealed a diet-independent, 5–13 % reduction in body mass in *schlank* knockdown flies (Fig. 1d), and also a loss of 10 % in triglyceride content (Fig. 1e). Negative controls ruled out effects on these traits from RU486 treatment alone (Additional file 1: Figures S3 and S4). These data make clear that *schlank* is of critical importance for the enhanced starvation resistance among DR flies, likely via remodeling of fat storage and catabolism in flies reared on this diet [36]. Lifespan assays suggested a potential role for schlank in DR-induced longevity, as knockdown of this gene shortened lifespan to a slightly greater degree in flies reared on the restricted diet (24 versus 19 % on AL; Additional file 1: Figure S5). Together, our discoveries of a role for *schlank* in nutrient-dependent changes in starvation resistance, as well as body weight, provide a first compelling validation of multi-phenotype association signal in the DGRP as a signpost for genes with true metabolic function.

**Fig. 2:**
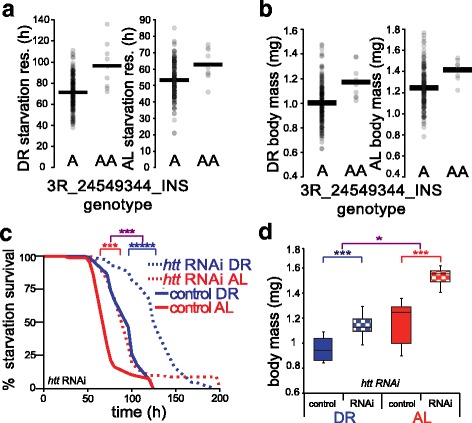
A metabolic function for fly *huntingtin* (*htt*). **a** and **b** Trait association to *htt* genotype in the DGRP. Data are as in Fig. 1a-b, except that an intronic indel in *htt* is analyzed. **c** and **d** Validation of *htt* function in laboratory strains. **c** Each trace reports survival of a strain expressing a GAL4 activator under the *daughterless* promoter and harboring an *htt* RNAi construct (RNAi) or no RNAi transgene (*control*), pre-treated for 10 days on the indicated diet and then switched to acute starvation media. **d** Each column reports body mass per fly of the indicated strain, with genotypes as in **c**. (*n* = 5 populations of 15 flies per condition). Symbols and abbreviations are as in Fig. 1. For strain details see Additional file 1: Table S6

### A metabolic role for fly *htt*

As a second case study for functional confirmation of our cross-phenotype mapping results, we focused on *htt*, the *Drosophila* huntingtin gene. In our survey of DGRP lines, a single-base insertion in an intron of fly *htt* reached significance for joint association to body mass and starvation resistance, in animals on the AL diet (Table 1), and a similar effect was discernable upon manual inspection in DR flies (Fig. 2a-b). A metabolic role for fly *htt* would be consistent with the symptoms of human Huntington’s patients, who, even on prescribed diets of thousands of excess calories a day, often fail to maintain body mass [50, 51]. To verify the dependence of fly nutrient responsive traits on *htt*, we bred a line with constitutive, ubiquitous expression of an *htt* RNAi construct, and confirmed knockdown in this strain by qRT-PCR (Additional file 1: Figure S2). This *htt* knockdown line exhibited marked improvements in starvation resistance relative to isogenic controls, in DR flies and, to a lesser degree, in animals reared on the AL diet (Fig. 2c). Knockdown of *htt* also conferred robust increases in body mass, with a slightly stronger effect in animals on AL food than on DR (24 versus 22 %, respectively; Fig. 2d), but had no significant effect on triglyceride content (Additional file 1: Figure S4). To test *htt*’s role in longevity while avoiding the potential for knockdown effects during development, we bred flies expressing drug-inducible, ubiquitous *htt* RNAi, and induced the transgene only after eclosion, confirming knockdown by qRT-PCR (Additional file 1: Figure S2). The resulting *htt* knockdown in adult animals conferred a 9 % lifespan extension on the AL diet and decreased lifespan in DR animals by 5 % relative to isogenic controls, effects that could not be attributed to inducer treatment alone (Additional file 1: Figure S5). These data make clear that reduced *htt* expression can alter longevity and promote starvation resistance and increase body mass, likely through mechanisms that do not involve changes to steady-state triglyceride levels. As nutrient restriction enhanced some of these effects, mitigated others, and had no detectable effect on the natural variant originally emerging in our association scan, *htt* likely participates in a complex relationship with other dietary response regulators.

### Common variants associated with metabolic traits

As an additional strategy to improve mapping power in the DGRP, we developed a scheme for analysis of polymorphisms at which the minor allele was very common. We expected that common variants mapped to a given trait might only represent a fraction of its full genetic architecture, but we considered this restriction a justifiable trade-off in light of the expected high power in association tests of common variants where the number of observations among individuals bearing the minor allele is maximized. For a first evaluation of this strategy, we applied our linear model association test framework for a given trait, including genotype and gene-by-diet interaction effects, to the 734,670 markers in the DGRP with minor allele frequency ≥25 %. This genome-scale scan uncovered significant, diet-independent association between genotype at *nimb3* and acute starvation resistance, as we had observed in scans of variants at lower allele frequencies; we also detected association to body mass at the uncharacterized gene *CG43921* (Table 1). Gene-by-diet association was significant between whole-body glucose measurements and a locus encompassing the cleavage and polyadenylation factor *pic* and the uncharacterized gene *CG7966* (Table 1). These loci explained up to 18 % of the variance in their respective traits (Table 1). Functional genomic tests revealed enrichment in association to starvation resistance among genes categories annotated in actin filament production (Additional file 1: Table S5). In the latter, top-scoring genes were *Arp2*, *Arpc2*, and *SCAR* (association *p* = 0.0008, 0.001, and 0.003 respectively), which have roles in the development of neural processes and axonal defasciculation [52, 53]. Also emerging from functional genomic tests was an enrichment in association to starvation resistance among heterophilic cell-cell adhesion genes (Additional file 1: Table S5); these included *Notch* (association *p* = 0.006), among whose many biological roles is that of axon guidance [54], and “beaten path” genes such as *beat*-*Ib*, *beat*-*IIIb*, and *beat*-*Va* (association *p* = 0.00001, *p* = 0.00008, *p* = 0.0002 respectively), which are involved in motor neuron defasciculation [55]. These results suggest that variants acting to tune development of the nervous system, if they modulate feeding or physical activity, could have downstream influences on metabolism [56].

### *CG43921*/*heavyweight* is a novel determinant of body mass

**Fig. 3:**
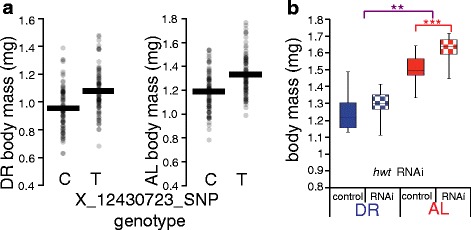
*CG43921*/*heavyweight* is a novel determinant of body mass. **a** DGRP trait association with *heavyweight* (*hwt*) genotype. Data are as in Fig. 1a-b except that an intronic variant in *hwt* is analyzed. **b** Validation of *hwt* function in laboratory strains. Each column reports body mass per fly of a strain expressing a GAL4 activator under the *daughterless* promoter and harboring an *hwt* RNAi construct (RNAi) or no RNAi transgene (control) (*n* = 2 populations of 15 flies per condition). Symbols and abbreviations are as in Fig. 1. For strain details see Additional file 1: Table S6

For a validation of a top-scoring locus from our association tests of common variants, we focused on the uncharacterized gene *CG43921*, in which an intronic polymorphism associated with body mass across DGRP lines in a diet-independent manner (Table 1 and Fig. 3a). To verify the dependence of fly body mass on *CG43921*, we bred a line with constitutive expression of a *CG43921* RNAi construct under a ubiquitous, constitutive driver, and we confirmed knockdown in this strain by qRT-PCR (Additional file 1: Figure S2). Knockdown of *CG43921* by this strategy increased body mass by 9 % relative to isogenic controls in flies reared on the AL diet, with no significant effect in DR animals (Fig. 3b). We did not find significant changes in triglyceride levels (Additional file 1: Figure S4) or lifespan (Additional file 1: Figure S5) and we observed only minor extension of starvation resistance (Additional file 1: Figure S6) in *CG43921* knockdown flies. These data implicate *CG43921* primarily in the control of body mass, the trait that showed the strongest association signal, and as such we give this gene the common name *heavyweight* (*hwt*).

### Diet-dependent metabolic function of *rdgA*

Paralleling our analyses of low-frequency variants, we used the set of common variants in the DGRP as input into tests for cross-phenotype associations, analyzing each pair of our phenotypes on each diet. Two significant single-locus mapping results emerged from analysis of flies reared on DR. Genotype at *CG43462*, a suspected determinant of fly lifespan [57], was associated with starvation resistance and triglyceride levels; the diacylglycerol kinase gene *rdgA* (Table 1), whose mammalian homologs have been implicated in diabetes and obesity risk [58, 59] and which also modulates fly lifespan [60], associated with body mass and starvation resistance. Functional genomic tests detected enriched association, in our joint analysis of starvation resistance and body mass, in two sets of genes annotated in alcohol catabolism (Additional file 1: Table S5). Top-scoring genes in these categories were three sphingomyelin phosphodiesterases (*CG15533*, *CG15534*, and *CG3376*; association *p* = 0.0099, 0.0012, and 0.0013, respectively) and two phosphatidylinositol phosphatases (*Plip* and *Pten*, association *p* = 0.0022 and 0.0056, respectively), highlighting the potential for variants throughout phospholipid pathways that could perturb metabolism in the DGRP.

**Fig. 4:**
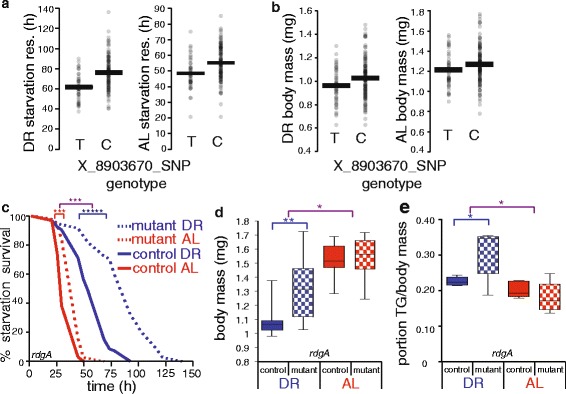
The diacylglycerol kinase *rdgA* governs body mass, starvation resistance, and lifespan. **a** and **b** Trait association to *rdgA* genotype in the DGRP. Data are as in Fig. 1a-b, except that an intronic variant in *rdgA* is analyzed. **c**-**e** Validation of *rdgA* function in laboratory strains. **c** Each trace reports survival of a strain harboring a transposon insertion in *rdgA* (mutant) or isogenic wild-type control, pre-treated for 10 days on the indicated diet and then switched to acute starvation media. **d** Each column reports body mass per fly of the indicated strain, with genotypes as in **c** (*n* = 2 populations of 15 flies per condition). **e** Each column reports whole body triglyceride levels normalized to body mass in the indicated strain, with genotypes as in **c** (*n* = 1 population of 15 flies per condition). Symbols and abbreviations are as in Fig. 1. For strain details see Additional file 1: Table S6

We chose *rdgA* for an additional validation case study, on the strength of previous evidence that it interacts with the TOR pathway and modulates lifespan [60]. The natural variant segregating in the DGRP in *rdgA* was associated with stronger effects in animals reared on the DR diet than on AL (Fig. 4a and b), though this diet dependence did not reach significance in our starvation resistance GWAS. We reasoned that disruption of *rdgA* might affect metabolic characters in a diet-specific manner. To test this, we first phenotyped flies bearing a transposon insertion in *rdgA* [61]. Relative to background-matched controls, these mutants survived acute starvation for 51 % longer after rearing on the DR diet, with a more modest 16 % increase after pre-treatment on the AL diet (Fig. 4c). *rdgA* mutants also exhibited an increase in mass, with an effect significantly stronger in DR flies than those on AL food (24 and 3 %, respectively; Fig. 4d). Consistent with the gain in body mass on DR, there was a significant 12 % increase in triglyceride levels in *rdgA* mutant flies relative to controls on DR but not AL (Fig. 4e). To investigate the role of *rdgA* in lifespan, we used a drug-inducible RNAi construct, triggering knockdown only in adulthood. Mirroring results of a previous study in flies reared on a single diet [60], we found that neuron-specific knockdown of *rdgA* was sufficient for lifespan extension; however, our data revealed this effect to be most pronounced during DR (9 % extension), and almost undetectable on the AL diet (Additional file 1: Figure S5). We conclude that laboratory-induced perturbations to *rdgA* impact starvation resistance, body mass, and lifespan. The striking diet dependence of effects of these mutations, as with natural polymorphisms at the locus, reflect a likely role for *rdgA* in nutrient response, as expected given its interactions with TOR signaling [60].

## Discussion

A direct consequence of obesity is the increased probability of age-related pathologies, including diabetes, cardiovascular disease, neurodegenerative disorders and cancer [1]. Conversely, fasting and dietary restriction prolong lifespan and delay the onset of chronic diseases [29, 62, 63]. The genes that underlie these effects are prime targets for pro-healthspan drugs and personalized diets, and natural variation mapping strategies are well suited for expedient screens to discover these factors. In this work, we have implemented several strategies for highly-powered GWAS in the fly, including a focus on homologs of genes involved in human disease and common variants, and tests of a given variant for joint relationships to multiple traits in parallel. Our work also provides a first window onto gene-by-diet interactions in the DGRP. In total, our association scans of common alleles and disease gene homologs have implicated 17 variants, in 12 genes, in nutrient response traits. And we identified groups of genes of common function with enriched association to metabolic traits, although formally, nuanced statistical biases would need to be ruled out as the potential source for any such pathway signal [64].

Among the single-locus GWAS hits validated in our study, three have confirmed or hypothesized roles in phospholipid signaling and lipid metabolism. *rdgA* encodes a diacylglycerol kinase that catalyzes the formation of phosphatidic acid, a phospholipid with pleiotropic signaling effects [60, 65]. Our discovery of *rdgA* as a regulator of body mass, starvation resistance, and lifespan dovetails with the remarkable pleiotropy of this gene, including roles in taste [66] and smell [67] as well as phototransduction [68], sound [69], temperature perception [70], and lifespan [60]. Under one plausible model, *rdgA*’s main link to body mass and nutrient response could be in the control of taste preference and/or feeding behavior. Alternatively, *rdgA*’s metabolic role could involve its known link to TOR signaling [60, 65, 71], plausibly via regulation of macroautophagy in the fat body [72] or global regulation of translation in response to stress [73]. The latter would be consistent with our finding that *rdgA* mutants are only long-lived when on a restricted diet. Another of our validated GWAS hit loci, the ceramide synthase *schlank* (homologous to the yeast lifespan gene *LAG1* [47]), is known to control larval body size and fat accumulation [46]. Compromised lipid storage is therefore a potential proximal cause for the drop in body mass, starvation resistance, and lifespan we observe upon *schlank* knockdown in adult flies. The latter effects could involve the redirection of biosynthetic resources to other lipid pathways as ceramide and sphingolipid biosynthesis is blocked, since *schlank* loss of function is known to perturb levels of a number of lipid species [46]. However, it is tempting to speculate that *schlank* could act as a master metabolic regulator, in light of its ubiquitous expression and the evidence for ceramide as a mediator of apoptosis [74, 75] and MAPKKK/JNK mediated stress-response signaling [74, 76, 77] in the fly. A third gene on which we focus here, *CG43921* (which we have named *heavyweight*), is of unknown biochemical function but is annotated with a FYVE domain which may bind phosphatidylinositol 3-phosphate (PI3P) [78], as well as a src homology 2 domain [79]. Future work will be necessary to establish whether *rdgA*, *schlank*, and *hwt*, and the cohort of additional phospholipid metabolic genes in which we detected enriched association signal (Additional file 1: Table S5), indeed exert effects on body mass and starvation resistance via phospholipid signaling. A particularly compelling model would invoke phospholipid regulation of the insulin pathway, to modulate apoptosis and cell growth [80] and/or glucose homeostasis [81–84].

Our results also reveal a novel metabolic function for fly *huntingtin*, reminiscent of the weight loss seen in human Huntington’s patients [50, 51] and in some mouse models of the disease [85–88]. The molecular-genetic origin of these phenotypes is a subject of ongoing debate, since in principle it could manifest from either a toxic gain-of-function in the mutant protein or a loss of wildtype huntingtin function [51, 86, 89]. As we observed dramatic increases in body size and starvation resistance in *htt* knockdown flies, our study provides a first compelling rationale for refuting the hypothesis that weight loss results from huntingtin loss of function. Our data leaves open the question of how native *htt* acts as a negative regulator of body mass and starvation resistance. Conceivably, its metabolic function could be mediated by circulating leptin and adiponectin [85, 89] and/or IGF-1 [89], which are perturbed by Huntington’s disease mutations in mammals. In the face of the early lethality of mouse *huntingtin* nulls [90] and the developmental defects in *huntingtin* knockdown zebrafish [91], expedient invertebrate models are sorely needed in the field. Our analyses of fly *htt* lay the groundwork for tests of mechanistic hypotheses centered around this key human disease gene.

## Conclusions

We have conducted genome-wide scans for variants associated with starvation resistance, body mass and composition and their response to changing diets. We have validated the effects of *schlank*, *rdgA*, and *htt* on diet-dependent traits, and we have uncovered a panel of additional candidates for future analyses. Alongside these novel genetic discoveries, our experimental design sets a precedent for the use of gene-by-diet and cross-phenotype association tests in fruit fly GWAS.

## Methods

### DGRP phenotyping

DGRP lines were obtained from the Bloomington *Drosophila* Stock Center [92]. Fly husbandry and food were as previously described [36].

To measure starvation resistance for a given line, an isogenic population of 100 non-virgin female flies was reared on either DR (0.5 % yeast) or AL (5 % yeast) food from the day of eclosion, with 25 flies per vial. After 10 days, flies were switched to starvation media (agar providing water but containing no nutrients), and deaths of flies were counted three times daily until all flies died; we then tabulated the average time to death across this isogenic population for use in statistical genetics analyses below. One biological replicate (one batch of 100 animals) was assayed for 159 DGRP strains and two biological replicates (two batches of 100 animals assayed starting on two different days) for another 12 strains.

For body mass, glucose and triglyceride measurements for a given line, an isogenic population of 15 seven day old non-virgin female flies was weighed in groups of 3 flies and then homogenized in 200 μl PBS and frozen. In contrast to the constitutive RNAi experiments, for adult-induced *schlank* RNAi we sampled schlank flies at 14 days after eclosion to allow enough time to observe a change in body weight and body composition. This whole-fly homogenate was subjected to two technical replicates of both the StanBio Triglycerides LiquiColor® Test (Cat# 2100-225) and the Glucose Liqui-UV® Test (Cat# 1060-500). The measurements were normalized to body mass [36]. One biological replicate (one batch of 15 animals) was assayed for 88 DGRP strains and two biological replicates (two batches of 15 animals each assayed on each of 2 days) for another 93 strains.

We filtered out cases in which variation between replicates for a given DGRP strain was >20-fold greater than the median intrastrain variation (among all our measurements, only triglyceride assays on nine DGRP strains failed to meet this filter) and subjected all remaining measurements to genetic analysis as detailed below.

### Heritability

Broad-sense heritabilities in Additional file 1: Table S2 were calculated for each trait as (σ^2^_total_ - σ^2^_intrastrain_)/ σ^2^_total_, using only the DGRP lines for which replicate measurements were carried out. For σ^2^_total_ for a given trait, we calculated the variance across all phenotype measurements for all such lines. Separately, for a given DGRP line we calculated the variance in a given trait across biological replicates; we then calculated the average of these variances across lines as our measure of σ^2^_intrastrain_.

### Genotype filtering

We used DGRP release 2 genotypes and FlyBase R5 coordinates for gene annotations [17, 93]. For the genome-wide scan of low-frequency variants, we used all DGRP polymorphic positions with minor allele frequency ≥5 % (1,932,643 total). For analysis of human disease orthologs, we subjected the set of FlyBase genes with human homologs [44] to a homolog search via DIOPT-DIST [94, 95] to find best-match human-*Drosophila* homologs, and then filtered for human genes with entries in the Online Mendelian Inheritance in Man database [42]. All DGRP polymorphisms with minor allele frequency ≥5 % (172,637 total) within 1 kb of a homologous gene model were used in these OMIM homolog association tests. We did not filter variants based on the coding status of the polymorphic locus. For the genome-wide scan of common variants, we used all DGRP markers with minor allele frequency ≥25 % (734,670 total).

### Single-trait association tests

For a given trait and marker, we used the complete cohort of average phenotype measurements across all replicates, from flies of all DGRP lines reared on both diets, as input into an association test via ordinary least squares regression using the statsmodels module in Python [96]. The linear model was phenotype = β_1_ x genotype + β_2_ x diet + β_3_ x genotype x diet + intercept. Nominal *p*-values denoted as “genotype” in Table 1 report the probability that β_1_ = 0, and those denoted as “interaction” report the probability that β_3_ = 0. To avoid the potential for false positives at a given nominal cutoff owing to *p*-value inflation, we calculated false discovery rates via permutation as follows: for a given permutation *i*, we randomized phenotype values across DGRP lines, retaining the true diet assignment, and on this permuted data set we carried out single-trait association tests for each marker in turn as above. We counted the number of markers *n* ^*i*^ that scored above a given *p*-value threshold *t*. We tabulated the false discovery rate at *t* as the ratio between the average *n* ^*i*^ across ten permutations and the number of markers called at *t* in the real data.

### Cross-phenotype association tests

We carried out cross-phenotype association tests for starvation resistance and body mass; starvation resistance and triglyceride levels; and starvation resistance and glucose levels as follows. We used the phenotype measurements for a given trait pair from flies of all DGRP lines reared on a given diet, and genotype at a given marker, as input into the R package SMAT [41]. False discovery rate estimates were calculated via permutation as above. Because triglyceride and glucose level measurements were each normalized by body mass for a given DGRP line, these phenotypes were not independent and therefore we did not subject any pair among them to cross-phenotype association testing.

Because our cross-phenotype association analysis involved testing over multiple trait pairs (Additional file 1: Table S4), we assessed the empirical false discovery rate (FDR) across the entire set of tests at a given nominal SMAT *p*-value threshold *t* as follows. We tabulated the number of loci *n*_*true*_ with SMAT significance at or exceeding *t* in at least one trait pair. Next, a permuted assignment of genotype to strain was used as input into a SMAT calculation for each trait pair in turn, yielding the number of loci *n*_*perm*_ with SMAT significance at or exceeding *t* in at least one trait pair for this permutation. We took the ratio between the average of *n*_*perm*_, across 10 permutations, and *n*_*true*_ as the multiple testing-corrected FDR across trait pairs. At *t* = 1.27E-08, corresponding to the SMAT *p*-value attained in the real data for our least significant SMAT hit, *rdgA* (Table 1), this procedure resulted in an estimated FDR of 12 %. Therefore our SMAT hits have in the worst case a 12 % multiple-testing corrected FDR. In the best case, the top scoring polymorphism in *schlank* returned *p* = 4.44E-16, a level that has a 2 % multiple-testing corrected FDR.

### Percent variance explained by mapped loci

For a given trait, we calculated the proportion of the variance across DGRP lines explained by a given mapped locus as the R^2^ from the model phenotype = β_1_ x genotype + intercept, fit via regression as above using measurements from all lines on both diets. The results serve as an upper bound on the proportion of variance explained, due to the Beavis effect [97, 98]. For a given locus mapped in cross-phenotype association tests, we carried out regression and calculated R^2^ as above for each of the two traits, and in Table 1 the greater of the two is reported.

### Functional genomic analysis

We tested for enrichment of association signal among gene ontology (GO) biological processes as follows. Given the results from a single-trait or cross-phenotype association scan across markers as above, we first tabulated the single best-scoring marker for every gene tested. We then used the ranked list of these genes as input into GOglm, which tests for GO term enrichment in a ranked list without arbitrary thresholding into significant and insignificant genes, and corrects for gene length effects [37]. Only GO terms with >10 members with association results were considered for analysis. The resulting enrichment significance estimates were corrected for multiple testing with the Benjamini-Hochberg method. Annotation was from GOseq [99].

### Gene knockdown by RNAi

For a given candidate gene, we used functional validation strategies that enable the study of a line in which the gene is perturbed as a comparison with an isogenic control of the same background. For this purpose, for each gene we acquired a strain harboring a UAS-driven RNAi construct [100] from VDRC [101], or from the TRiP resource at the Bloomington *Drosophila* Stock Center [92], and we also acquired lines expressing a constitutive GAL4 driver [102] or an RU486-inducible GeneSwitch (GS) GAL4-human progesterone receptor fusion protein driver [103]. Strains used are listed in Additional file 1: Table S6. Drivers were checked for activity by mating to UAS-GFP transgene and assaying for GFP fluorescence in the progeny. For each knockdown experiment, five males of the UAS-RNAi line of interest were crossed to 20 virgin females of a GAL4 line. As negative controls for the latter, five males lacking the RNAi construct (background-matched to the RNAi lines) were crossed to 20 females of the same GAL4 line. In the case of *rdgA* we compared a previously characterized transposon insertion mutant [61] to a background-matched control. For each experiment, non-virgin female progeny were collected as day-old adults and phenotyped as described below.

### qRT-PCR analysis of RNAi target expression

To confirm RNAi-mediated knockdown in a given line in Additional file 1: Figure S2, total RNA was extracted from three frozen whole flies per condition via Zymo Quick RNA MiniPrep kit (R1054). For qRT-PCR, we used Superscript III Platinum SYBR Green One-Step qRT-PCT kit from Invitrogen (11736-051) and followed the manufacturer’s instructions with a Stratagene Mx3000P qPCR machine. We used a six point standard curve for each target gene to calibrate the relative abundance levels of the amplified mRNA in three technical replicate samples per condition. ROX dye was used to normalize for reaction volume loading.

### Phenotyping of knockdown and mutant strains

We used knockdown and mutant strains to test candidate genes’ roles in body mass, starvation resistance and lifespan. Genotypes of control and experimental strains are listed in Additional file 1: Table S6. For body mass and starvation experiments using GS drivers, at day 1 of adulthood, flies were sorted onto food containing either the GS inducer RU486, or ethanol as a control, and body mass or starvation was assayed 10 days later as for the DGRP strains (see above). To measure body mass, cohorts of 15 flies reared on a given diet were frozen and weighed at day 7 after eclosion for *htt* and *CG43921*, and day 14 for *schlank* and *rdgA*. For body mass, significance in a given diet was tested by *t*-test, and interaction between the genetic perturbation and diet was tested by ANOVA. Starvation resistance assays were performed as on DGRP strains (see above); each trace in main and supplementary figures reports a representative experiment from among 2–5 biological replicates of 100 non-virgin female flies per condition. Lifespan assays used 200 non-virgin female flies per condition, 25 flies per vial, changing media every 2 days. For lifespan assays all knockdowns were induced only in adult flies. Each trace in Additional file 1: Figure S5 reports a representative experiment from among 1–5 biological replicates of 100–200 flies per condition. Survival curves for starvation resistance and lifespan were analyzed with the Cox proportional hazards method implemented in the R package “survival”.

## Additional file

Additional file 1: Table S1. Correlation between phenotypes in DGRP lines raised on two different diets. **Table S2.** Broad-sense heritabilities of metabolic traits. **Table S3.** GO terms enriched for association signal in scans of variants with allele frequency ≥5 %. **Table S4.** Phenotype pairs tested via SMAT. **Table S5.** GO terms enriched for association signal in scans of variants with allele frequency ≥25 %. **Table S6.** Strains and crosses used. **Figure S1.** Variation in metabolic traits and nutrient response across the DGRP. In a given bar chart, each bar reports the mean of the indicated phenotype in an isogenic population of one DGRP line reared on the indicated diet. Strains are ordered along the *x*-axis by AL diet phenotype. The right hand panels report the same data formatted as a scatterplot. (A), Resistance to acute starvation after rearing adults for 10 days on the indicated diet. (B), Body mass. (C), Whole body triglyceride (TG) content, normalized by body mass. (D), Whole body glucose content, normalized by body mass. a.u., dimensionless arbitrary units. AL, *ad libitum* diet; DR, dietary restriction. **Figure S2.** Decreased expression of *schlank*, *htt*, *hwt*, and *rdgA* upon RNAi. In the first, second, fourth, and sixth panels, each bar reports qRT-PCR measurements of expression of the indicated gene, in a line reared on the indicated diet and harboring both the GeneSwitch (GS) activator and a construct for RNAi of the indicated gene under the control of the indicated driver (*da*, *daughterless*; *elav*, *embryonic lethal*, *abnormal vision*), treated with the GS inducer RU486 (+) or a vehicle control (-); in a given diet, each expression measurement is normalized to that from the respective control-treated animals. In the third and fifth panels, each bar reports qRT-PCR measurements of expression of the indicated gene in a strain expressing an RNAi construct for the indicated gene regulated by GAL4 under the *daughterless* promoter (RNAi) or a background-matched control (Ctrl) with no RNAi construct; in a given diet, each expression measurement is normalized to that from the respective control animals. **Figure S3.** The GeneSwitch inducer RU486 has no effect on starvation resistance or body mass. (A), Each trace reports survival of flies expressing *daughterless* promoter (*da*)-driven GAL4-Gene Switch (GS) that do not harbor an RNAi construct, with drug treatment (dotted lines) or ethanol vehicle treatment (solid lines) starting from eclosion (*n* = 2 biological replicates of 100 flies per condition). (B), Each column reports the distribution of body mass per fly of the no-RNAi control strain harboring the *da*-GS driver, with genotypes and treatments as in (A) (*n* = 2 populations of 15 flies per condition). The checkered boxes indicate addition of the RU486 inducer drug. Top, middle, and bottom horizontal bars of a given vertical box denote the respective quartiles across batches and technical replicates, and the top and bottom short horizontal bars report minimum and maximum, respectively. No changes here were significant by *t*-test at alpha = 0.05. AL, *ad libitum* diet; DR, dietary restriction. For strain details see Additional file 1: Table S6. **Figure S4.** The GeneSwitch inducer RU486 has no effect on triglyceride levels in the absence of an RNAi construct, and *htt* and *hwt* knockdown does not detectably impact triglycerides. (A), Each bar reports whole-body triglyceride levels in a strain expressing a ubiquitous *da*-GeneSwitch driver and no RNAi transgene, treated with the GeneSwitch inducer RU486 (“+ drug”) or with ethanol alone (“- drug”). (B) and (C), Each bar reports whole-body triglyceride levels in a strain expressing constitutive *da*-GAL4 either with an RNAi construct for the indicated gene (“RNAi”) or without any RNAi construct for the indicated gene (“control”) (*n* = 1 population of 15 flies per condition). For strain details see Additional file 1: Table S6. AL, animals reared on the *ad libitum* diet; DR, dietary restriction. Horizontal lines in each boxplot indicate quartiles across technical replicates, and the top and bottom short horizontal bars report minimum and maximum, respectively. No changes here were significant by *t*-test at alpha = 0.05. **Figure S5.** Lifespan effects of *schlank*, *htt*, *hwt* and *rdgA* knockdown. In each panel, the right-hand plot reports survival of flies harboring an RNAi construct for one gene regulated by *Act5C*-driven GAL4-GeneSwitch treated with the GeneSwitch inducer RU486 (“+ drug”) or with ethanol alone (“no drug”), and the left-hand plot is for a background-matched control without an RNAi transgene. Drug or vehicle treatment was started at eclosion. (A), *schlank* RNAi. (B), *htt* RNAi. (C), *hwt* RNAi. (D), *rdgA* RNAi. AL, *ad libitum* diet; DR, dietary restriction. *, *p* < 0.05, **, *p* < 0.01, ***, *p* < 10^-3^, ****, p <10^-7^, *****, *p* < 10^-15^. Red asterisks denote significance of the effect of the genetic perturbation in animals reared on AL food, blue denotes significance in animals on DR food, and purple denotes significance of the interaction between diet and genetic perturbation. For strain details see Additional file 1: Table S6. **Figure S6.** *hwt* knockdown modestly affects resistance to acute starvation. Each trace reports survival, under acute starvation, of flies harboring a GAL4 regulator under a *daughterless* promoter and either a GAL4-regulated RNAi construct for *hwt* (dotted lines) or a matched control without a transgene (solid line). AL, animals reared before starvation treatment on the *ad libitum* diet; DR, dietary restriction. For strain details see Additional file 1: Table S6. *, *p* < 0.05, **, *p* < 0.01. (DOCX 2 mb)

## Abbreviations

AL: *Ad libitum* diet. 5 % yeast media
DGRP: *Drosophila* Genetic Reference Panel
DR: Dietary restriction. 0.5 % yeast media
FDR: False discovery rate
GO: Gene ontology
GS: GeneSwitch RU486-inducible GAL4 transactivator
GWAS: Genome-wide association study
RNAi: RNA interference
UAS: Upstream activation sequence
