# Supplementary material for: Cross-phenotype association tests uncover genes mediating nutrient response in Drosophila
Source: BMC Genomics. 2016 Nov 4;17:867. doi: 10.1186/s12864-016-3137-9 (PMC5095962; doi:10.1186/s12864-016-3137-9)
Supplement: Additional file 1: Table S1. — Correlation between phenotypes in DGRP lines raised on two different diets. Table S2. Broad-sense heritabilities of metabolic traits. Table S3. GO terms enriched for association signal in scans of variants with allele frequency ≥5 %. Table S4. Phenotype pairs tested via SMAT. Table S5. GO terms enriched for association signal in scans of variants with allele frequency ≥25 %. Table S6. Strains and crosses used. Figure S1. Variation in metabolic traits and nutrient response across the DGRP. In a given bar chart, each bar reports the mean of the indicated phenotype in an isogenic population of one DGRP line reared on the indicated diet. Strains are ordered along the x-axis by AL diet phenotype. The right hand panels report the same data formatted as a scatterplot. (A), Resistance to acute starvation after rearing adults for 10 days on the indicated diet. (B), Body mass. (C), Whole body triglyceride (TG) content, normalized by body mass. (D), Whole body glucose content, normalized by body mass. a.u., dimensionless arbitrary units. AL, ad libitum diet; DR, dietary restriction. Figure S2. Decreased expression of schlank, htt, hwt, and rdgA upon RNAi. In the first, second, fourth, and sixth panels, each bar reports qRT-PCR measurements of expression of the indicated gene, in a line reared on the indicated diet and harboring both the GeneSwitch (GS) activator and a construct for RNAi of the indicated gene under the control of the indicated driver (da, daughterless; elav, embryonic lethal, abnormal vision), treated with the GS inducer RU486 (+) or a vehicle control (-); in a given diet, each expression measurement is normalized to that from the respective control-treated animals. In the third and fifth panels, each bar reports qRT-PCR measurements of expression of the indicated gene in a strain expressing an RNAi construct for the indicated gene regulated by GAL4 under the daughterless promoter (RNAi) or a background-matched control (Ctrl) with no RNAi construct; in a given [file 12864_2016_3137_MOESM1_ESM.docx]

**SUPPLEMENTAL INFORMATION**

**Table S1. Correlation between phenotypes in DGRP lines raised on two different diets.**

| **phenotype** | **R^2^*** |
| --- | --- |
| glucose levels | 0.53 |
| triglyceride levels | 0.54 |
| starvation resistance | 0.69 |
| body mass | 0.68 |

*Correlation coefficient between measurements of the indicated phenotype in a given line raised on restricted and *ad libitum* diets.

**Table S2. Broad-sense heritabilities of metabolic traits.**

|  |  | **diet*** |  |
| --- | --- | --- | --- |
| **phenotype** | **DR** | **AL** | **DR/AL** |
| starvation | 0.673 | 0.927 | 0.756 |
| glucose | 0.540 | 0.471 | 0.399 |
| triglycerides | 0.512 | 0.534 | 0.364 |
| body mass | 0.537 | 0.487 | 0.214 |

*Values in the first two columns report broad-sense heritabilities across DGRP lines raised on the indicated diet; the third column reports broad-sense heritabilities of the ratio of measurements of the indicated phenotype in animals reared on the two diets. DR, dietary restriction; AL, *ad libitum*.

**Table S3. GO terms enriched for association signal in scans of variants with allele frequency ≥5%.**

| **phenotype, test^*^** | **GO term^^^** | **GO name^^^** | ***p°*** |
| --- | --- | --- | --- |
| glucose, genotype | GO:0048800 | antennal morphogenesis | 1E-3 |
| body mass, genotype | GO:0003008 | system process | 3E-4 |
| body mass, genotype | GO:0071804 | cellular potassium ion transport | 3E-4 |
| body mass, genotype | GO:0071805 | potassium ion transmembrane transport | 3E-4 |
| body mass, genotype | GO:0050877 | neurological system process | 2E-3 |
| body mass, genotype | GO:0007600 | sensory perception | 5E-3 |
| body mass, genotype | GO:0050954 | sensory perception of mechanical stimulus | 0.03 |
| triglycerides, genotype | GO:0061327 | anterior Malpighian tubule development | 0.049 |
| starvation resistance and glucose on AL, SMAT | GO:0007419 | ventral cord development | 0.01 |

*The single phenotype whose measurements in animals reared on both diets were tested in a linear model with terms for diet, genotype, and the interaction between the two; or the pair of phenotypes whose measurements in animals reared on the indicated diet were tested via SMAT. DR, dietary restriction; AL, *ad libitum*.

**^^^**Gene Ontology term enriched for the indicated association signal in the genomic scan of markers with minor allele frequency ≥5%.

°Benjamini-Hochberg-corrected *p* value for enrichment. Results are only shown for terms with *p* < 0.05.

**Table S4. Phenotype pairs tested via SMAT.**

| **Phenotype 1^*^** | **Phenotype 2^*^** | **MAF^^^** | **best FDR^** |
| --- | --- | --- | --- |
| AL starvation resistance | AL body mass | 5% | 10% |
| AL starvation resistance | AL body mass | 25% | 53% |
| AL starvation resistance | AL glucose | 5% | 25% |
| AL starvation resistance | AL glucose | 25% | 87% |
| AL starvation resistance | AL triglycerides | 5% | 0% |
| AL starvation resistance | AL triglycerides | 25% | 30% |
| DR starvation resistance | DR body mass | 5% | 0% |
| DR starvation resistance | DR body mass | 25% | 10% |
| DR starvation resistance | DR glucose | 5% | 100% |
| AL starvation resistance | AL glucose | 25% | 40% |
| DR starvation resistance | DR triglycerides | 5% | 55% |
| DR starvation resistance | DR triglycerides | 25% | 10% |

*The pair of phenotypes whose measurements in animals reared on the indicated diet were tested via the scaled multiple-phenotype association test (SMAT). DR, dietary restriction; AL, *ad libitum*.

*Lowest minor allele frequency at which variants were considered for analysis. For scans of variants at frequency of ≥5%, only human disease orthologs were tested.

^False discovery rate at the single locus showing the strongest signal in the indicated scan.

**Table S5. GO terms enriched for association signal in scans of variants with allele frequency ≥25%.**

| **phenotype, test*** | **GO term^^^** | **GO name^^^** | ***p°*** |
| --- | --- | --- | --- |
| starvation resistance, genotype | GO:0031334 | positive regulation of protein complex assembly | 1E-4 |
| starvation resistance, genotype | GO:0032273 | positive regulation of protein polymerization | 9E-4 |
| starvation resistance, genotype | GO:0030838 | positive regulation of actin filament polymerization | 1E-3 |
| starvation resistance, genotype | GO:0045010 | actin nucleation | 0.04 |
| starvation resistance, interaction | GO:0007157 | heterophilic cell-cell adhesion | 5E-3 |
| starvation resistance and body mass on AL, SMAT | GO:0046164 | alcohol catabolic process | 0.02 |
| starvation resistance and body mass on AL, SMAT | GO:1901616 | organic hydroxy compound catabolic process | 0.02 |
| starvation resistance and glucose on AL, SMAT | GO:0007157 | heterophilic cell-cell adhesion | 0.03 |
| starvation resistance and glucose on AL, SMAT | GO:0032273 | positive regulation of protein polymerization | 0.03 |
| starvation resistance and glucose on AL, SMAT | GO:0030838 | positive regulation of actin filament polymerization | 0.03 |

*The single phenotype whose measurements in animals reared on both diets were tested in a linear model with terms for diet, genotype, and the interaction between the two; or the pair of phenotypes whose measurements in animals reared on the indicated diet were tested for cross-phenotype association by the scaled multiple-phenotype association test (SMAT). DR, dietary restriction; AL, *ad libitum*.

**^^^**Gene Ontology term enriched for the indicated association signal in the scan across markers with minor allele frequency ≥25%.

°Benjamini-Hochberg-corrected *p* value for enrichment. Results are only shown for terms with *p* < 0.05.

**Table S6. Strains and crosses used.**

| **A** | **Figures** | **trait** | **gene of interest** | | **experimental strain*** | | | | **control strain^^^** | | |
| --- | --- | --- | --- | --- | --- | --- | --- | --- | --- | --- | --- |
|  | 1C, S2, S3A | starvation resistance | *schlank* | | da-GS X BL29340 RNAi | | | | da-GS X TRiP control | | |
|  | 1D, S2, S3B | body mass | *schlank* | | da-GS X BL29340 RNAi | | | | da-GS X TRiP control | | |
|  | 1E, S4 | triglyceride | *schlank* | | da-GS X BL29340 RNAi | | | | da-GS X TRiP control | | |
|  | S2, S5 | lifespan | *schlank* | | Act5C-GS X BL29340 RNAi | | | | Act5C GS X TRiP control | | |
|  | 2C, S2 | starvation resistance | *htt* | | da-Gal4 x BL44550 RNAi | | | | da-Gal4 xTRiP control | | |
|  | 2D, S2 | body mass | *htt* | | da-Gal4 x BL44550 RNAi | | | | da-Gal4 xTRiP control | | |
|  | S4 | triglyceride | *htt* | | da-Gal4 x BL44550 RNAi | | | | da-Gal4 xTRiP control | | |
|  | S2, S5 | lifespan | *htt* | | Act5C-GS X BL44550 RNAi | | | | Act5C-GS X TRiP control | | |
|  | S2, S6 | starvation resistance | *CG43921/ hwt* | | da-Gal4 x v50598 RNAi | | | | da-Gal4 x W1118 | | |
|  | 3B, S2 | body mass | *CG43921/ hwt* | | da-Gal4 x v50598 RNAi | | | | da-Gal4 x W1118 | | |
|  | S4 | triglyceride | *CG43921/ hwt* | | da-Gal4 x v50598 RNAi | | | | da-Gal4 x W1118 | | |
|  | S2, S5 | lifespan | *CG43921/ hwt* | | Act5C-GS X v50598 RNAi | | | | Act5C-GS X W1118 | | |
|  | 4C | starvation resistance | *rdgA* | | BL20320 rdgA mutant | | | | W1118 | | |
|  | 4D | body mass | *rdgA* | | BL20320 rdgA mutant | | | | W1118 | | |
|  | 4E | triglyceride | *rdgA* | | BL20320 rdgA mutant | | | | W1118 | | |
|  | S2, S5 | lifespan | *rdgA* | | elav-GS X v102909 RNAi | | | | elav-GS X W1118 | | |
|  |  |  | |  | | |  | | |  | |
| **B** | **strain name** | **genotype** | | | | **source** | | **description** | | | **accession number(s)** |
|  | w1118 | w^1118^ | | | | Kapahi lab stock | | wild-type background | | | 5905 |
|  | TRiP control | y^1^ sc^*^ v^1^; P{VALIUM20-mCherry}attP2 | | | | Bloomington *Drosophila* Stock Center | | TRiP background control | | | 35785 |
|  | Actin5C-GS | P{w[+mC]=Act5C(FRT.y[+])GAL4.Switch.PR}X, y[1] w[*] backcrossed to local w^1118^ | | | | Bloomington *Drosophila* Stock Center | | inducible ubiquitous driver stock | | | 9431 |
|  | elav-GS | w^1118^ ; P{w[+mC]=elav-Switch.O}GSG301 backcrossed to local w^1118^ | | | | Bloomington *Drosophila* Stock Center | | inducible neuronal driver stock | | | 43642 |
|  | da-GAL4 | w*; P{w+mW.hs=GAL4-da.G32}UH1 | | | | Bloomington *Drosophila* Stock Center | | ubiquitous driver stock | | | 55851 |
|  | da-GS | w^1118^ ; P{ da-GSGAL4}  backcrossed to local w^1118^ | | | | Tricoire *et al.* 2009 referenced in main text | | inducible ubiquitous driver stock | | | NA |
|  | UAS-GFP | y* w*; P{w[+mC]=UAS-2xEGFP}AH3 | | | | Bloomington *Drosophila* Stock Center | | fluorescent protein for driver checking | | | 6658 |
|  | BL29340 | y^1^ v^1^; P{TRiP.JF02502}attP2 | | | | Bloomington *Drosophila* Stock Center | | *schlank* RNAi | | | 29340 |
|  | BL44550 | y^1^ sc^*^ v^1^; P{TRiP.HMS02845}attP2 | | | | Bloomington *Drosophila* Stock Center | | *htt* RNAi | | | 44550 |
|  | v50598 | w^1118^; P{GD15014}v50598 | | | | Vienna *Drosophila* Resource Center | | *CG43921* RNAi | | | 50598 |
|  | BL20320 | y^1^ w^67c23^ P{EPgy2}rdgA^EY11543^ | | | | Bloomington *Drosophila* Stock Center | | *rdgA* transposon insertion mutant | | | 20320 |
|  | v102909 | P{KK113317}VIE-260B | | | | Vienna *Drosophila* Resource Center | | *rdgA* RNAi | | | 102909 |

*For knockdown experiments, parents crossed to generate the strain denoted “RNAi” in the indicated figure panel; for experiments with mutants, identifier of the strain denoted “mutant” in the indicated panel. Strain details are in panel B.

^^^For knockdown experiments, parents crossed to generate the strain denoted “control” in the indicated figure panel; for experiments with mutants, identifier of the strain denoted “control” in the indicated panel. Strain details are in panel B.

**
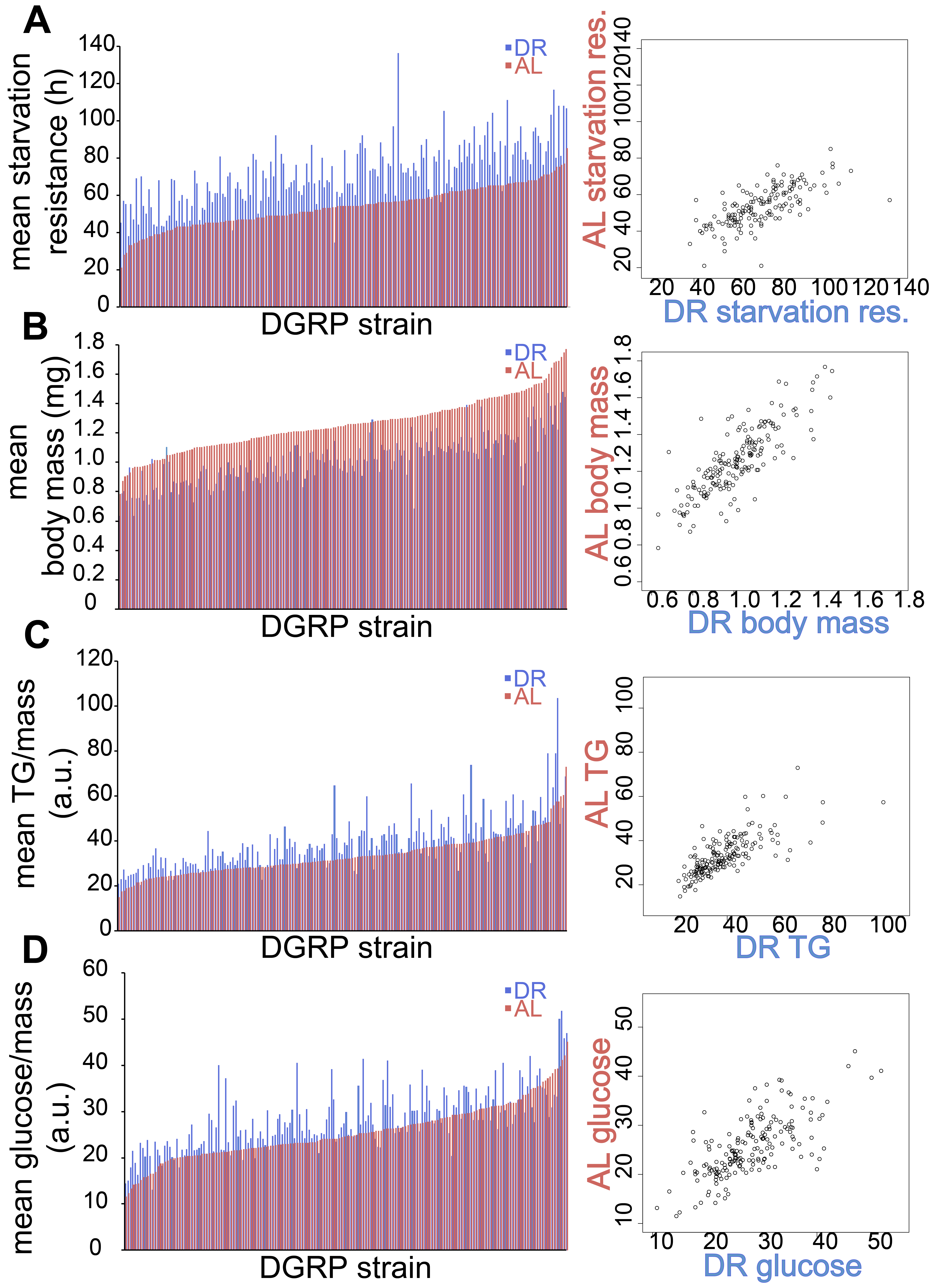
**

**Figure S1. Variation in metabolic traits and nutrient response across the DGRP.** In a given bar chart, each bar reports the mean of the indicated phenotype in an isogenic population of one DGRP line reared on the indicated diet. Strains are ordered along the *x*-axis by AL diet phenotype. The right hand panels report the same data formatted as a scatterplot. (A), Resistance to acute starvation after rearing adults for 10 days on the indicated diet. (B), Body mass. (C), Whole body triglyceride (TG) content, normalized by body mass. (D), Whole body glucose content, normalized by body mass. a.u., dimensionless arbitrary units. AL, *ad libitum* diet; DR, dietary restriction.

**
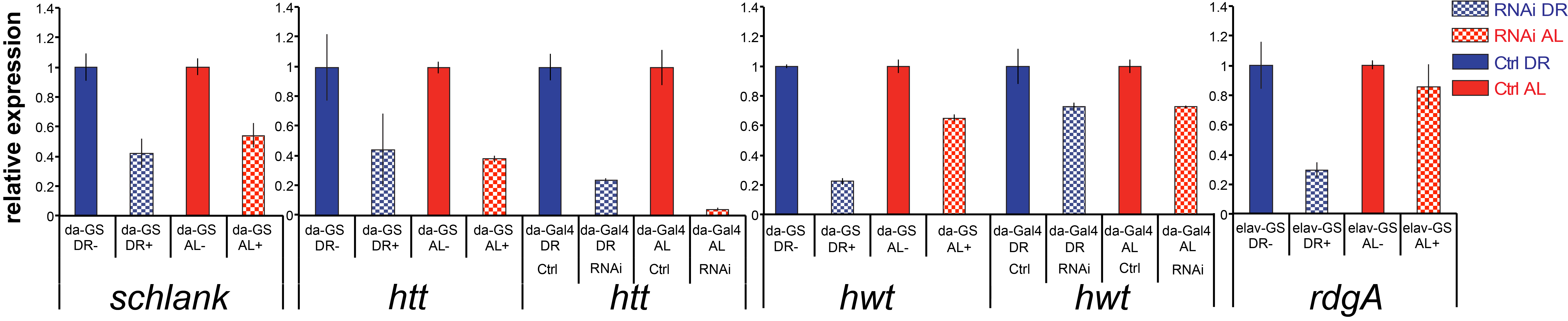
**

**Figure S2. Decreased expression of *schlank*, *htt, hwt,* and *rdgA* upon RNAi.** In the first, second, fourth, and sixth panels, each bar reports qRT-PCR measurements of expression of the indicated gene, in a line reared on the indicated diet and harboring both the GeneSwitch (GS) activator and a construct for RNAi of the indicated gene under the control of the indicated driver (*da, daughterless; elav, embryonic lethal, abnormal vision*), treated with the GS inducer RU486 (+) or a vehicle control (-); in a given diet, each expression measurement is normalized to that from the respective control-treated animals. In the third and fifth panels, each bar reports qRT-PCR measurements of expression of the indicated gene in a strain expressing an RNAi construct for the indicated gene regulated by GAL4 under the *daughterless* promoter (RNAi) or a background-matched control (Ctrl) with no RNAi construct; in a given diet, each expression measurement is normalized to that from the respective control animals.


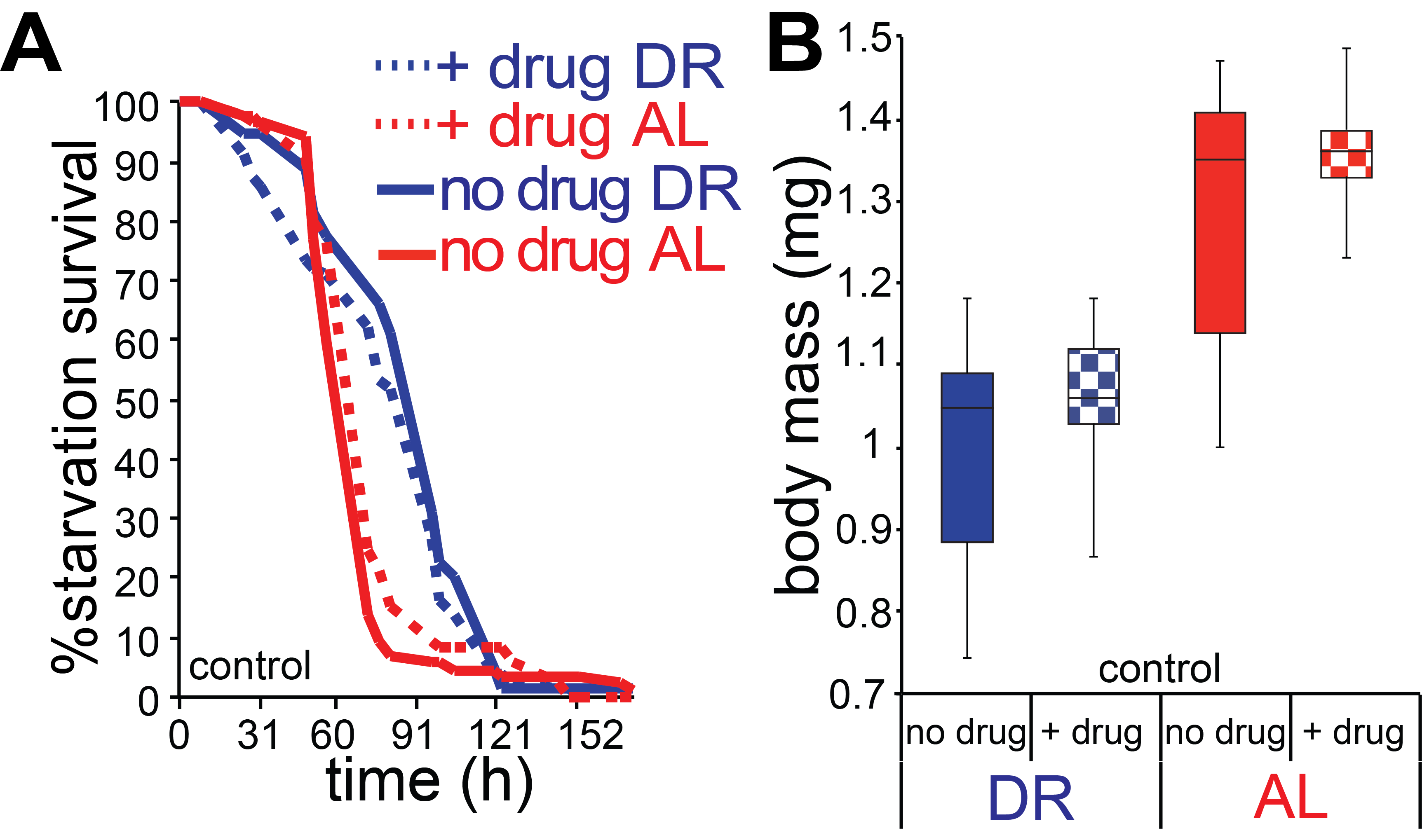


**Figure S3. The GeneSwitch inducer RU486 has no effect on starvation resistance or body mass.** (A), Each trace reports survival of flies expressing *daughterless* promoter (*da*)-driven GAL4-Gene Switch (GS) that do not harbor an RNAi construct, with drug treatment (dotted lines) or ethanol vehicle treatment (solid lines) starting from eclosion (*n* = 2 biological replicates of 100 flies per condition). (B), Each column reports the distribution of body mass per fly of the no-RNAi control strain harboring the *da*-GS driver, with genotypes and treatments as in (A) (*n* = 2 populations of 15 flies per condition). The checkered boxes indicate addition of the RU486 inducer drug. Top, middle, and bottom horizontal bars of a given vertical box denote the respective quartiles across batches and technical replicates, and the top and bottom short horizontal bars report minimum and maximum, respectively. No changes here were significant by t-test at alpha=0.05. AL, *ad libitum* diet; DR, dietary restriction. For strain details see Table S6.

**
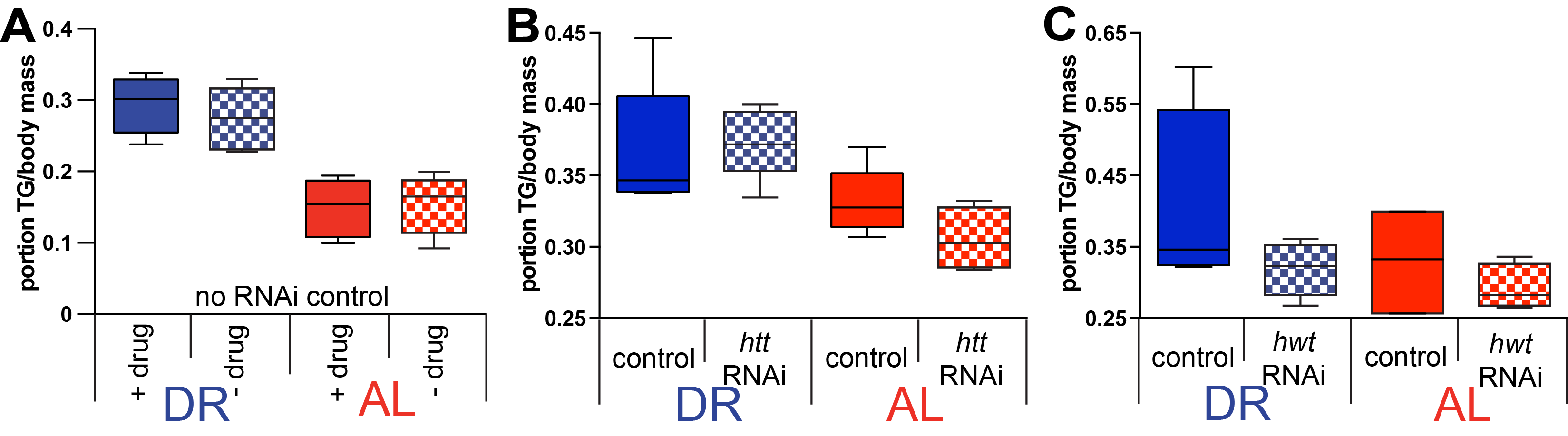
**

**Figure S4. The GeneSwitch inducer RU486 has no effect on triglyceride levels in the absence of an RNAi construct, and *htt* and *hwt* knockdown does not detectably impact triglycerides.** (A), Each bar reports whole-body triglyceride levels in a strain expressing a ubiquitous da-GeneSwitch driver and no RNAi transgene, treated with the GeneSwitch inducer RU486 (“+ drug”) or with ethanol alone (“- drug”). (B) and (C), Each bar reports whole-body triglyceride levels in a strain expressing constitutive *da*-GAL4 either with an RNAi construct for the indicated gene (“RNAi”) or without any RNAi construct for the indicated gene (“control”) (*n* = 1 population of 15 flies per condition). For strain details see Table S6. AL, animals reared on the *ad libitum* diet; DR, dietary restriction. Horizontal lines in each boxplot indicate quartiles across technical replicates, and the top and bottom short horizontal bars report minimum and maximum, respectively. No changes here were significant by t-test at alpha=0.05.


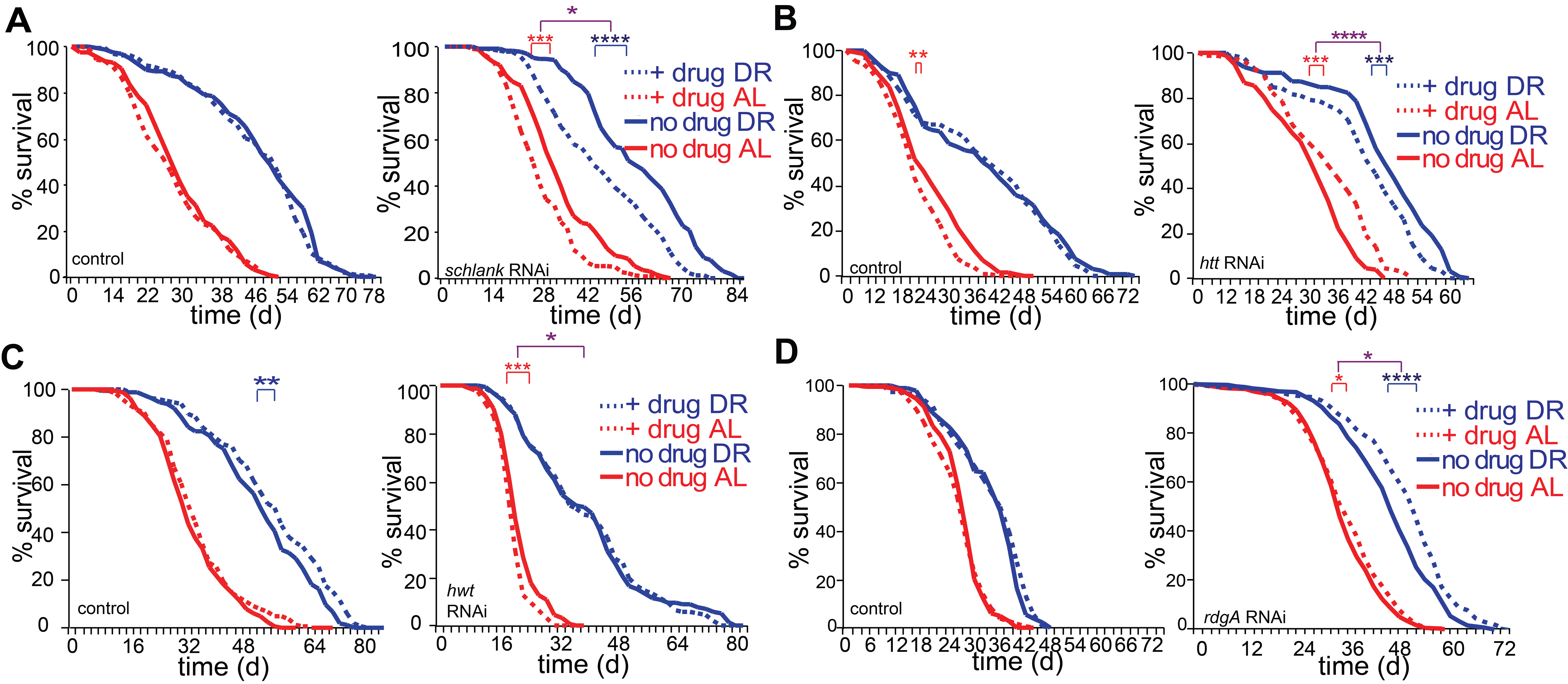


**Figure S5. Lifespan effects of *schlank*, *htt*, *hwt* and *rdgA* knockdown.** In each panel, the right-hand plot reports survival of flies harboring an RNAi construct for one gene regulated by *Act5C*-driven GAL4-GeneSwitch treated with the GeneSwitch inducer RU486 (“+ drug”) or with ethanol alone (“no drug”), and the left-hand plot is for a background-matched control without an RNAi transgene. Drug or vehicle treatment was started at eclosion. (A), *schlank* RNAi. (B), *htt* RNAi. (C), *hwt* RNAi. (D), *rdgA* RNAi. AL, *ad libitum* diet; DR, dietary restriction. *, *p* < 0.05, **, *p* < 0.01, ***, *p* < 10^-3^, ****, p <10^-7^, *****, *p* < 10^-15^. Red asterisks denote significance of the effect of the genetic perturbation in animals reared on AL food, blue denotes significance in animals on DR food, and purple denotes significance of the interaction between diet and genetic perturbation. For strain details see Table S6.


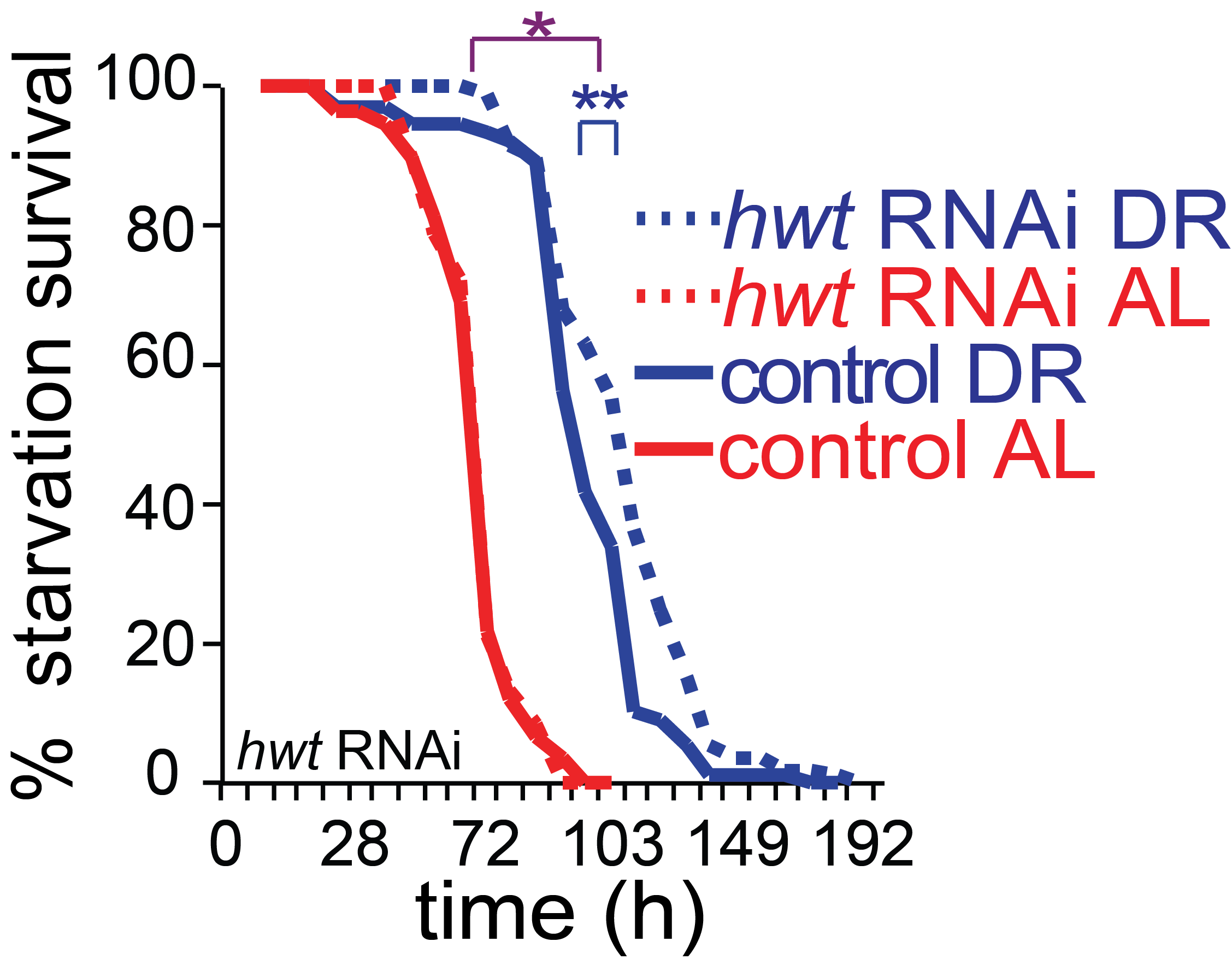


**Figure S6. *hwt* knockdown modestly affects resistance to acute starvation.** Each trace reports survival, under acute starvation, of flies harboring a GAL4 regulator under a *daughterless* promoter and either a GAL4-regulated RNAi construct for *hwt* (dotted lines) or a matched control without a transgene (solid line). AL, animals reared before starvation treatment on the *ad libitum* diet; DR, dietary restriction. For strain details see Table S6. *, *p* < 0.05, **, *p* < 0.01.
